# 371. Adding sputum and saliva to nasopharyngeal swab samples for PCR detection of Respiratory Syncytial Virus in adults hospitalized with acute respiratory illness may double case detection

**DOI:** 10.1093/ofid/ofac492.449

**Published:** 2022-12-15

**Authors:** Julio A Ramirez, Ruth Carrico, Ashley M Wilde, Alan Junkins, Stephen Furmanek, Thomas R Chandler, Paul S Schulz, Robin Hubler, Paula Peyrani, Paula Peyrani, Sonali Trivedi, Sonal Uppal, Qing Liu, Bradford D Gessner, Elizabeth Begier

**Affiliations:** Norton Healthcare, Louisville, Kentucky; Norton Healthcare, Louisville, Kentucky; Norton Healthcare, Louisville, Kentucky; Norton Healthcare, Louisville, Kentucky; Norton Healthcare, Louisville, Kentucky; Norton Healthcare, Louisville, Kentucky; Norton Healthcare, Louisville, Kentucky; Pfizer Inc., Collegeville, Pennsylvania; Pfizer, Inc, Collegeville, Pennsylvania; Pfizer, Inc, Collegeville, Pennsylvania; Pfizer, Collegeville, Pennsylvania; Pfizer, Collegeville, Pennsylvania; Pfizer Inc., Collegeville, Pennsylvania; Pfizer Biopharma Group, Collegeville, Pennsylvania; Pfizer Vaccines, Dublin, Dublin, Ireland

## Abstract

**Background:**

In hospitalized patients, nasopharyngeal (NP) swabs are the most common samples obtained for Respiratory Syncytial Virus (RSV) PCR testing. However, adding sputum is known to increase diagnostic yield, and saliva has been successfully used for viral respiratory infection diagnosis. We sought to compare RSV prevalence detected by PCR testing of NP swab alone versus NP swab plus saliva and sputum in adult patients hospitalized with acute respiratory illness (ARI).

**Methods:**

This ongoing, prospective cohort study enrolled patients aged ≥40 years hospitalized for ARI in 4 hospitals in Louisville, Kentucky (Season 1: 27 Dec 21 – 1 Apr 22). NP swab, saliva, and sputum samples were obtained at enrollment or scavenged from standard-of-care specimens (all collected ≤3 days of admission), and PCR tested with Luminex ARIES FluA/B/RSV platform. We produced Venn diagrams of RSV positive samples by sample type for all patients and restricted to those with all 3 sample types. RSV prevalence for NP swab alone was calculated as number of patients with RSV-positive NP swabs divided by total number of patients tested. RSV prevalence by NP swab plus saliva and sputum was calculated as number of patients with RSV-positive NP swab, saliva, or sputum samples divided by total number of patients tested.

**Results:**

We enrolled 653 patients and collected NP swabs (100% of patients), saliva (96%), and sputum (43% overall and 93% of the 303 sputum-producing patients). Among all patients, 28 patients tested RSV positive (Figure 1A), and when restricted to those with all 3 samples (Figure 1B), 14 tested positive. The overall cohort’s RSV prevalence by NP swab alone was 1.8% (12/653) and by NP swab plus saliva and/or sputum was 4.3% (28/653): 2.33 times higher with addition of saliva and sputum samples. Among patients with all 3 specimen types, the RSV prevalence increase was the same, and none were positive by NP swab only.

**Figure 1.**
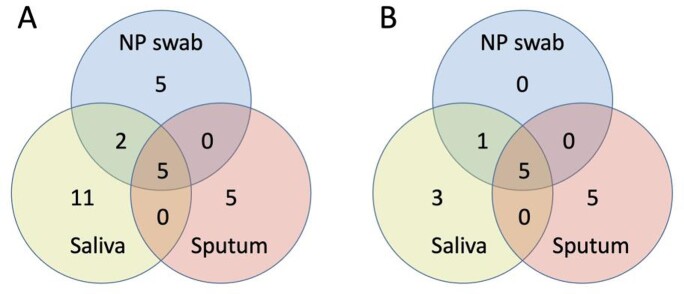
Venn diagrams of positive RSV PCR tests

(Left) A. Positive RSV PCR tests for 653 patients in overall cohort (Right) B. Positive RSV PCR tests for 275 patients with all 3 samples obtained.

**Conclusion:**

RSV was most commonly detected in saliva samples. Current standard-of-care utilizing NP swab for RSV PCR testing appears to underestimate true RSV prevalence in hospitalized adult patients with ARI by more than 2-fold.

**Disclosures:**

**Alan Junkins, PhD, D(ABMM)**, Biomerieux: Advisor/Consultant **Paul S. Schulz, MD**, Gilead: Advisor/Consultant|Gilead: Grant/Research Support|Gilead: Honoraria|Merck: Advisor/Consultant|Merck: Grant/Research Support|Merck: Honoraria **Robin Hubler, MS**, Pfizer Inc.: Employee|Pfizer Inc.: Stocks/Bonds **Paula Peyrani, MD**, Pfizer, Inc: Employee|Pfizer, Inc: Employee|Pfizer, Inc: Stocks/Bonds|Pfizer, Inc: Stocks/Bonds **Paula Peyrani, MD**, Pfizer, Inc: Employee|Pfizer, Inc: Employee|Pfizer, Inc: Stocks/Bonds|Pfizer, Inc: Stocks/Bonds **Qing Liu, M.S.**, Pfizer Inc.: I am a full time employee of Pfizer and hold Pfizer stocks **Bradford D. Gessner, M.D., M.P.H.**, Pfizer Inc.: Employee|Pfizer Inc.: Stocks/Bonds **Elizabeth Begier, M.D., M.P.H.**, Pfizer: Employee|Pfizer: Stocks/Bonds.

